# Potential diagnostic markers and therapeutic targets for non-alcoholic fatty liver disease and ulcerative colitis based on bioinformatics analysis and machine learning

**DOI:** 10.3389/fmed.2024.1323859

**Published:** 2024-11-06

**Authors:** Zheng Luo, Cong Huang, Jilan Chen, Yunhui Chen, Hongya Yang, Qiaofeng Wu, Fating Lu, Tian E. Zhang

**Affiliations:** ^1^Chengdu University of Traditional Chinese Medicine, Chengdu, China; ^2^Key Biology Laboratory for TCM Viscera-Manifestation Research of Sichuan University, Chinese Medical Center of Chengdu University of TCM, Chengdu, China

**Keywords:** bioinformatics, non-alcoholic fatty liver disease, ulcerative colitis, machine learning, diagnosis, immune infiltration

## Abstract

**Background:**

Non-alcoholic fatty liver disease (NAFLD) and ulcerative colitis (UC) are two common health issues that have gained significant global attention. Previous studies have suggested a possible connection between NAFLD and UC, but the underlying pathophysiology remains unclear. This study investigates common genes, underlying pathogenesis mechanisms, identification of diagnostic markers applicable to both conditions, and exploration of potential therapeutic targets shared by NAFLD and UC.

**Methods:**

We obtained datasets for NAFLD and UC from the GEO database. The DEGs in the GSE89632 dataset of the NAFLD and GSE87466 of the UC dataset were analyzed. WGCNA, a powerful tool for identifying modules of highly correlated genes, was employed for both datasets. The DEGs of NAFLD and UC and the modular genes were then intersected to obtain shared genes. Functional enrichment analysis was conducted on these shared genes. Next, we utilize the STRING database to establish a PPI network. To enhance visualization, we employ Cytoscape software. Subsequently, the Cytohubba algorithm within Cytoscape was used to identify central genes. Diagnostic biomarkers were initially screened using LASSO regression and SVM methods. The diagnostic value of ROC curve analysis was assessed to detect diagnostic genes in both training and validation sets for NAFLD and UC. A nomogram was also developed to evaluate diagnostic efficacy. Additionally, we used the CIBERSORT algorithm to explore immune infiltration patterns in both NAFLD and UC samples. Finally, we investigated the correlation between hub gene expression, diagnostic gene expression, and immune infiltration levels.

**Results:**

We identified 34 shared genes that were found to be associated with both NAFLD and UC. These genes were subjected to enrichment analysis, which revealed significant enrichment in several pathways, including the IL-17 signaling pathway, Rheumatoid arthritis, and Chagas disease. One optimal candidate gene was selected through LASSO regression and SVM: CCL2. The ROC curve confirmed the presence of CCL2 in both the NAFLD and UC training sets and other validation sets. This finding was further validated using a nomogram in the validation set. Additionally, the expression levels of CCL2 for NAFLD and UC showed a significant correlation with immune cell infiltration.

**Conclusion:**

This study identified a gene (*CCL2*) as a biomarker for NAFLD and UC, which may actively participate in the progression of NAFLD and UC. This discovery holds significant implications for understanding the progression of these diseases and potentially developing more effective diagnostic and treatment strategies.

## 1 Introduction

Non-alcoholic fatty liver disease (NAFLD) is a prevalent condition characterized by the accumulation of fat in the liver cells, known as steatosis (1). NAFLD includes a range of diseases ranging from NAFL to NASH, which involves both fat accumulation and inflammation in the liver. NASH can progress to more severe forms of liver damage, including cirrhosis and HC. The global prevalence of NAFLD is estimated to be around 25%, making it one of the most common chronic liver diseases worldwide (2). One concerning aspect of NAFLD is its association with various extrahepatic complications. Research has shown that individuals with NAFLD have an increased risk of developing CVD, CKD, and T2DM (3).

Ulcerative colitis (UC), a chronic inflammatory bowel disease, caused by multiple reasons and mediated by abnormal immunity. The main manifestations are recurrent diarrhea, abdominal pain, and mucopurulent bloody stools (4). Looking toward future trends, it is estimated that there will be approximately 5 million cases of UC worldwide by 2023 (5). In recent years, more and more studies have pointed out that Irritable bowel syndrome (IBS) and IBD (including Crohn’s disease and UC) have partial overlap in clinical manifestations and pathophysiological mechanisms. For example, studies have found that IBS-like symptoms are not uncommon in patients with IBD, especially when IBD is in remission, and this overlap can lead to misdiagnosis and mistreatment (6, 7). In addition, intestinal microecological disorder, intestinal barrier dysfunction and low-grade chronic inflammation may be the common underlying pathological mechanisms (8). Due to the high prevalence of IBS, this greatly increases the prevalence of NAFLD due to intestinal causes (9).

Recent studies have highlighted the co-existence of NAFLD and UC (7). NAFLD is a prevalent condition among individuals with UC, affecting up to one-third of UC patients globally. Moreover, research has shown that UC patients are twice as likely to develop NAFLD compared to healthy individuals (8). In recent years, studies have also suggested a potential association between UC and NAFLD through the enteric-liver axis (10). Chronic intestinal inflammation may cause bacterial products (such as endotoxins) to enter the liver through the portal vein by disrupting the intestinal barrier function, thereby triggering an inflammatory response in the liver and promoting the occurrence and development of NAFLD (11, 12). In this complex process, the immune system’s involvement is equally important (13). Therefore, based on the complex crosstalk between two diseases, Identifying factors related to the co-development of two syndromes and identifying common pathophysiological pathways can represent diagnostic and treatment strategies, which is of great significance.

**Figure 1:**
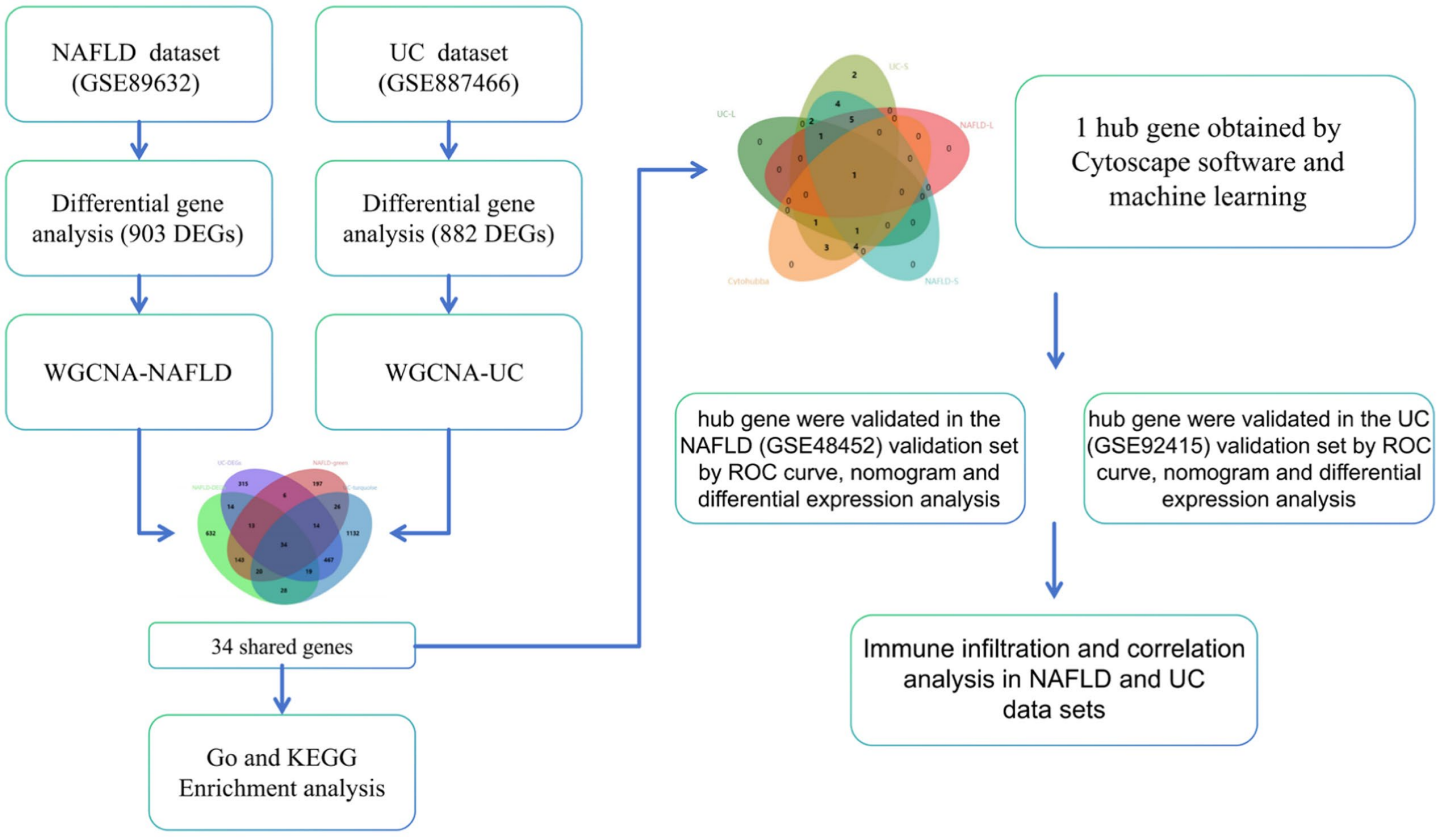
Detailed flowchart of research design.

Advancements in the field of bioinformatics have revolutionized our ability to study coexpressed genes that are associated with NAFLD and UC. These tools have opened up new opportunities for researchers to delve deeper into the molecular mechanisms underlying these diseases and identify potential targets for treatment (14). We analyzed the coexpressed genes of NAFLD and UC using bioinformatics tools. By examining common genes across both NAFLD and UC, we have identified diagnostic markers that could indicate disease presence or progression. Additionally, we provide valuable information about immune infiltration within both affected NAFLD and UC. This research has significant implications for improving patient outcomes through more targeted therapies explicitly tailored toward individuals with NAFLD and UC. The workflow can be seen in Figure 1.

## 2 Materials and methods

### 2.1 Data acquisition

Four datasets were downloaded from the GEO database (15). The GSE89632 (16) dataset comprises 24 HC patients and 39 NAFLD patients. The GSE87466 (17) dataset comprises 21 HC and 87 UC patients. Additionally, we also obtained the GSE48452 (18) dataset consisting of liver tissues from 32 NAFLD patients and 41 HC patients, as well as the GSE92415 (19) dataset consisting of liver tissues from 162 UC patients and 21 HC patients for external validation purposes. Details for the data sets were provided in Table 1.

**Table 1:** Detailed information of datasets used in this study.

ID	Organism	Platform	Normal vs. NAFLD	Type of samples
GSE89632	Human (samples of liver)	GPL14951	24 vs. 39 (62)	NAFLD
GSE87466	Human (Mucosal biopsy samples)	GPL13158	21 vs. 87 (108)	UC
GSE48452	Human (samples of liver)	GPL11532	41 vs. 32 (73)	NAFLD
GSE92415	Human (Mucosal biopsy samples)	GPL13158	21 vs. 162(183)	UC

### 2.2 Identification of DEGs

We utilized the “limma” R package to analyze the GSE89632 dataset and identify DEGs between the NAFLD and HC groups. The criteria for selecting DEGs were set at |log2 FC| ≥ 0.886 or 1 and p-adjust <0.05. To visualize the DEGs, a volcano plot was generated using the “ggplot2” package. Additionally, a heatmap was created to display the top 25 DEGs. Additionally, we applied the same methodology described above to analyze another dataset called GSE87466.

### 2.3 Weighted gene co-expression network analysis

The WGCNA method was utilized to identify functional modules. To ensure the accuracy of the analysis, only the top 25% of genes with the highest variation were chosen for further investigation. Next, to eliminate any ineligible genes and samples, the goodSamplesGenes function was employed, creating a scale-free coexpression network. Subsequently, the soft powers *β* were determined using the pickSoftThreshold function. A dynamic tree-cutting technique was applied to detect gene modules within the coexpression network. Additionally, these identified gene modules will be linked to clinical features. Finally, a Venn diagram on a bioinformatics platform (20) displayed the overlapping genes between NAFLD’s green module genes with DEGs and UC’s turquoise module genes with DEGs.

### 2.4 Functional enrichment analysis

We used GSEA with version 4.8.2 of the “clusterProfiler” package to identify KEGG pathways enriched by key genes in NAFLD and UC. Additionally, we conducted GO and KEGG enrichment analyses on the Metascape platform (21) to explore the functional biological roles of co-expressed genes. We applied a significance threshold of *p*-value <0.01 for these analyses. The results were then visualized using Sankey dot pathway enrichment diagrams on a bioinformatics platform.

### 2.5 Construction of PPI network and identification of hub genes

To analyze the interactions between commonly recognized genes, the string platform was used to construct PPI networks. Setting screening criteria with interaction scores exceeding 0.4 is significant. Next, Cytoscape software is utilized to visualize. We applied the cytoHubba plugin in Cytoscape, which calculates centrality measures based on maximal cliques within a network, allowing for identification of highly connected nodes or hub genes.

### 2.6 Machine learning for screening key genes and validation

The LASSO and the SVM algorithm were used to identify key genes in GSE89632 and GSE87466. Tw o independent datasets, GSE48452 and GSE92415, were used to validate the critical genes identified. The “glmnet” R package was employed for the LASSO logistic regression analysis. The smallest lambda value obtained from this analysis was considered optimal for predicting disease outcomes. SVM, powerful machine learning algorithms, and RFE that select a subset of relevant genes by iteratively eliminating less informative ones based on five-fold cross-validation were employed to acquire critical genes. The ROC curves were generated using the pROC package to evaluate diagnostic accuracy. The “Rms” package is utilized to construct a nomograph and assess its predictive capability through the calibration curve—box plots generated by ggplot2 visualized expression levels of diagnostic genes in different groups or conditions. Statistical significance was denoted as *p < 0.05 and **p < 0.01.

### 2.7 Immune infiltration analysis

The abundance of 22 subtypes of immune cells in NAFLD samples and UC samples was determined using the cibersort algorithm from the “IOBR” package in R software and the CIBERSOTRx platform.^1^ This analysis provided insights into the cellular composition of the immune microenvironment in both NAFLD and UC. Additionally, we performed Spearman correlation analysis to investigate potential relationships between hub genes and different subsets of immune cells.

## 3 Results

### 3.1 Screening of DEGs in NAFLD and UC datasets

**Figure 2:**
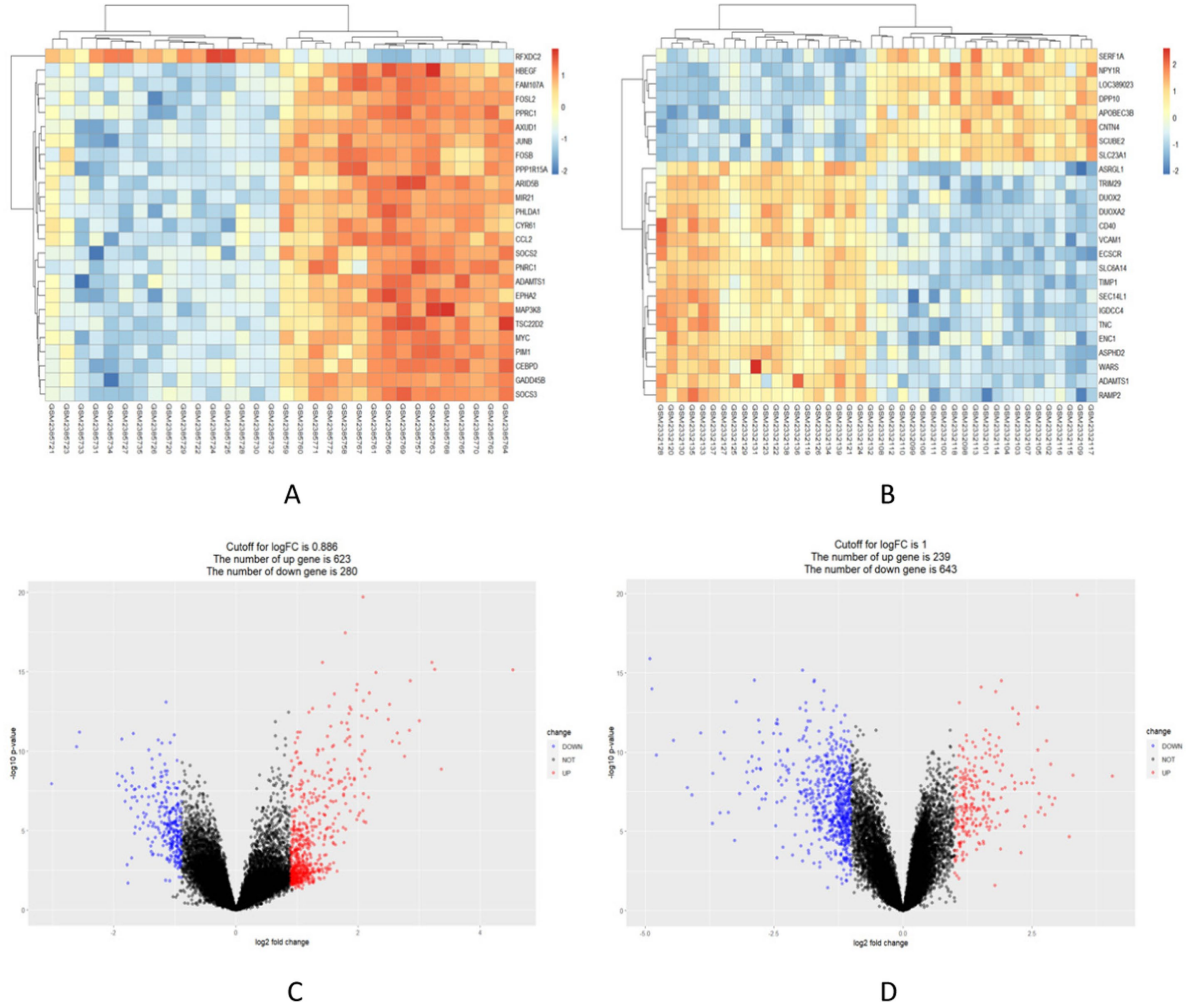
Identification of in the integrated NAFLD and UC datasets. (A,B) Red indicates upregulated DEGs, while blue indicates downregulated DEGs. (C,D) Red circles represent upregulated DEGs, while blue circles represent downregulated DEGs.

903 DEGs were found in the GSE89632 dataset (16 healthy individuals and 16 NAFLD patients were selected). Among these DEGs, 623 genes were upregulated, while 280 genes were down-regulated. The significance of these DEGs is further highlighted in Figure 2A, where the top 25 most significant DEGs are displayed as a heatmap. Figure 2C shows the DEGs by the volcano diagram. We obtained 882 UC-related DEGs in the GSE87466 dataset (21 healthy individuals and 21 patients with UC were selected). This analysis showed more downregulated genes (643) than upregulated ones (239). The heatmap and volcano diagram are shown in Figures 2B,D.

### 3.2 The co-expression modules in NAFLD and UC

**Figure 3:**
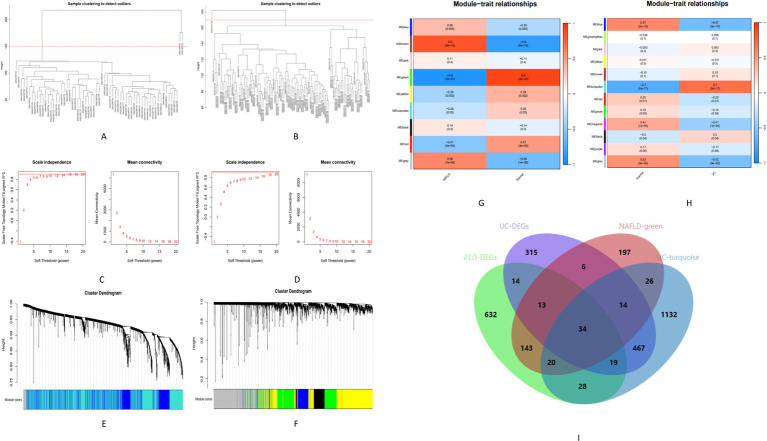
Identification of module genes in NAFLD and UC. (A,B) Clustering dendrogram showing the samples from NAFLD and UC. (C,D) The soft threshold (*β*) was determined to be 16 and 18, respectively, with a correlation coefficient of 0.9. (E,F) Each branch in the cluster diagram represents a gene, with different colors indicating a gene co-expression module. (G,H) Heatmap illustrating the relationships between modules and traits. (I) Venn diagram depicting four sets.

We used WGCNA to identify co-expressed gene profiles in the NAFLD (GSE89632) and UC datasets (GSE87466). After removing abnormal samples, we obtained clustering dendrograms for NAFLD and UC (Figures 3A,B). For the NAFLD dataset, a soft-threshold power *β* of 16 was chosen based on an R2 greater than 0.9, indicating scale independence (Figure 3C). Similarly, a soft-threshold power β of 18 for the UC dataset was chosen (Figure 3D). Using dynamic hybrid shearing, nine gene coexpression modules were produced for NAFLD, and 12 gene coexpression modules were produced for UC (Figures 3E,F). The correlation between these gene modules and NAFLD/Normal is depicted in Figure 3G. Notably, The first module, called the green module, consisted of 453 genes. We observed a robust correlation between this module and NAFLD. Likewise, the correlation between these gene modules and UC/Normal (Figure 3H). The second module, known as the turquoise module, contained 1740 genes. We discovered a significant correlation between this module and UC. Therefore, the green and turquoise modules were selected for subsequent analysis. Subsequently, the intersection of these four sets, including the DEGs of NAFLD, the DEGs of UC, the green module, and the turquoise, yielded 34 common genes (Figure 3I). Further research on these specific genes could provide valuable insights into understanding the underlying mechanisms linking these two conditions together.

### 3.3 Functional correlation analysis

**Figure 4:**
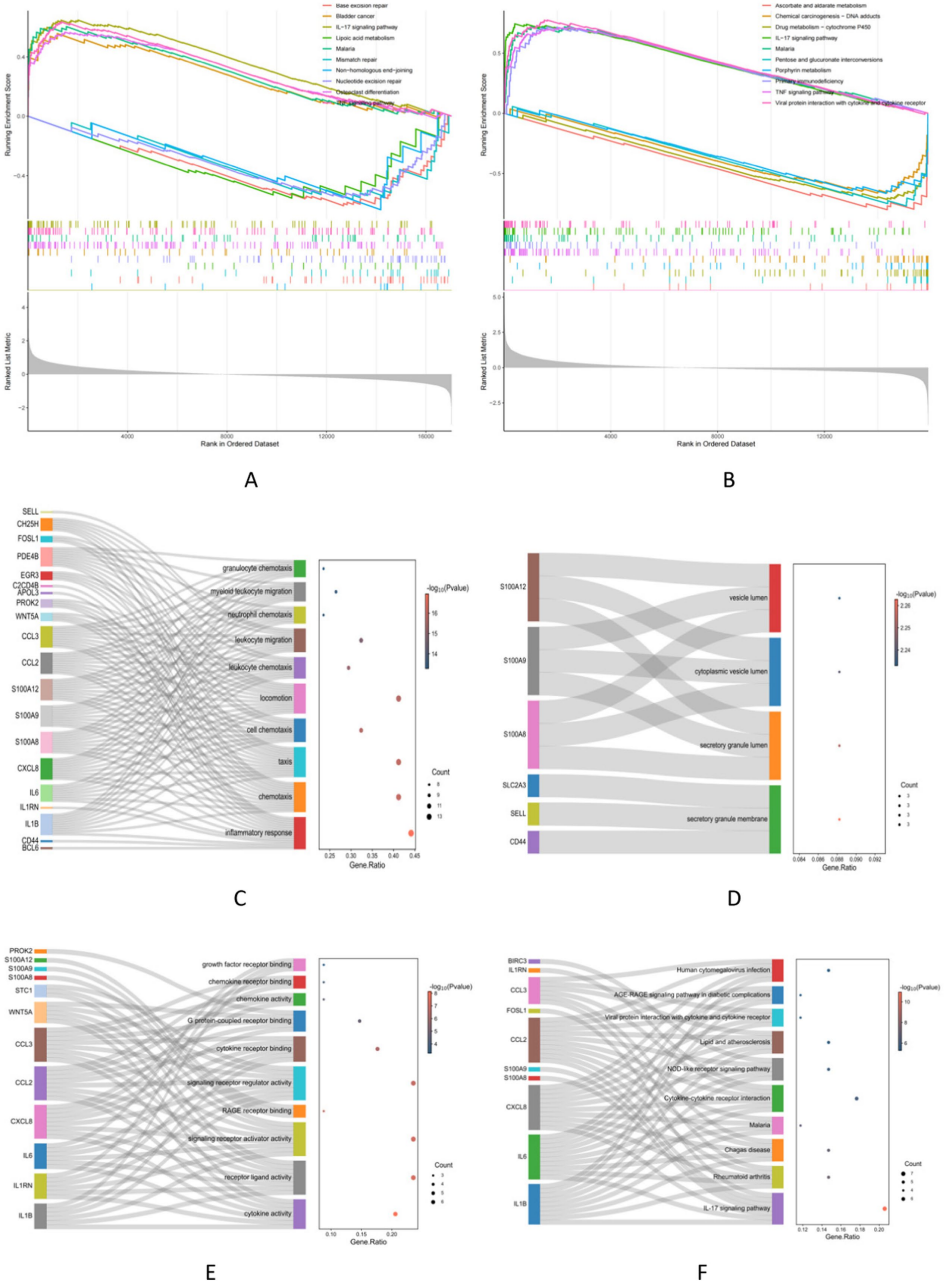
Functional correlation analysis. (A,B) The GSEA analysis results show the top 5 up and down groups of signaling pathways in NAFLD and UC. (C–E) The outcomes of GO enrichment analysis. (F) KEGG enrichment analysis reveals 10 pathways associated with key genes.

The GSEA analysis results revealed the top five in the NAFLD active UP group. These pathways included Bladder cancer, IL-17 signaling pathway, Osteoclast differentiation, Malaria, and TNF signaling pathway. The active down group included Base excision repair, Lipoic acid metabolism, Mismatch repair, Non-homologous end-joining, and Nucleotide excision repair (Figure 4A). Similarly, in the UC group, the active UP-enriched pathways were found to be the IL-17 signaling pathway, Malaria, Primary immunodeficiency, TNF signaling pathway, and Viral protein interaction with cytokine and cytokine receptor. The active down group included Ascorbate and alternate metabolism, Chemical carcinogenesis−DNA adducts, Drug metabolism-cytochrome P450, Pentose and glucuronate interconversions, and Porphyrin metabolism (Figure 4B). Next, With the metascape platform, further GO and KEGG enrichment analysis of critical genes showed their involvement in various BPs such as inflammatory response, chemotaxis, and taxis (Figure 4C). Regarding CCs, essential genes were principally associated with the secretory granule membrane, secretory granule lumen, and cytoplasmic vesicle lumen (Figure 4D). The MFs analysis indicated cytokine activity, receptor-ligand activity, and signaling receptor activator activity (Figure 4E). The essential genes’ top three significant KEGG pathways were enriched in the IL-17 signaling pathway, Rheumatoid arthritis, and Chagas disease (Figure 4F).

### 3.4 PPI network analysis and Core genes selection

**Figure 5:**
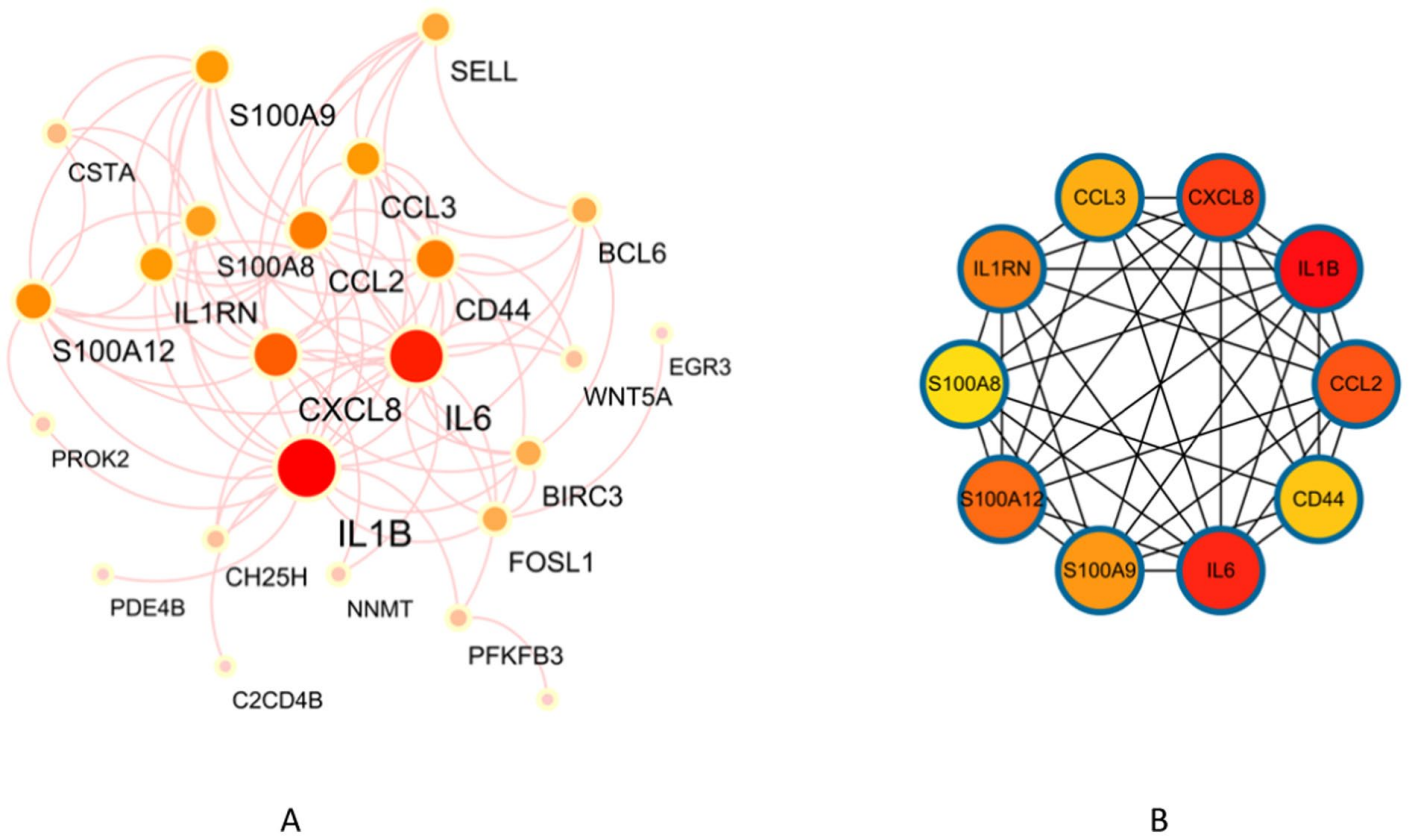
Identification of key genes in NAFLD and UC. (A) PPI network. (B) Co-expression of key genes in MCC algorithm by Cytoscape plugin cytoHubba.

A PPI network consisting of 34 protein targets was constructed using the STRING database. They were deleting disconnected nodes in the network. There were 24 nodes and 81 edges in Figure 5A. The cytoHubba plugin in Cytoscape was employed to score the importance of each gene based on their connectivity within the network. After applying this analysis method, the top 10 key genes with the highest node scores were identified, including *IL1B*, *IL6*, *CXCL8 (IL8)*, *CCL2*, *S100A12*, *IL1RN*, *S100A9*, *CCL3*, *CD44*, and *S100A8* (Figure 5B).

### 3.5 Machine learning for screening core genes and validation

**Figure 6:**
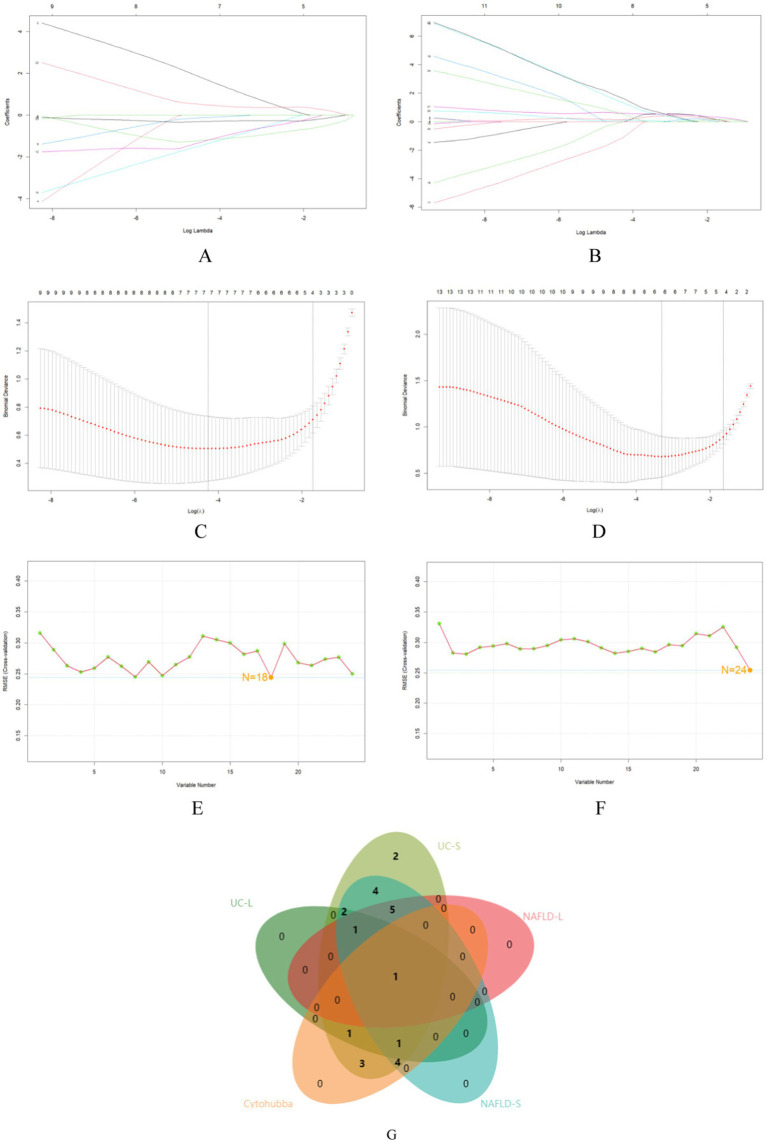
The use of machine-learning algorithms to screen for potential biomarkers of NAFLD with UC. (A,B) LASSO logistic regression used to analyze the core genes. (C,D) Five-fold cross-validation for turning parameter (*λ*) selection in the LASSO regression model. (E,F) SVM-RFE algorithm used to analyze the core genes. (G) Venn diagram of the intersection of five sets of core genes.

24 core genes were screened using the two machine learning. LASSO logistic regression identified 7 diagnostic core genes of NAFLD in the model. On the other hand, the SVM-RFE method identified a more extensive set of 18 diagnostic core genes for NAFLD (Figures 6A,C,E). LASSO logistic regression and SVM identified 6 and 24 diagnostic core genes, respectively, for UC (Figures 6B,D,F). These two diseases’ common diagnostic core genes are considered diagnostic markers for NAFLD with UC (Figure 6G). Finally, one diagnostic marker was obtained: *CCL2*.

**Figure 7:**
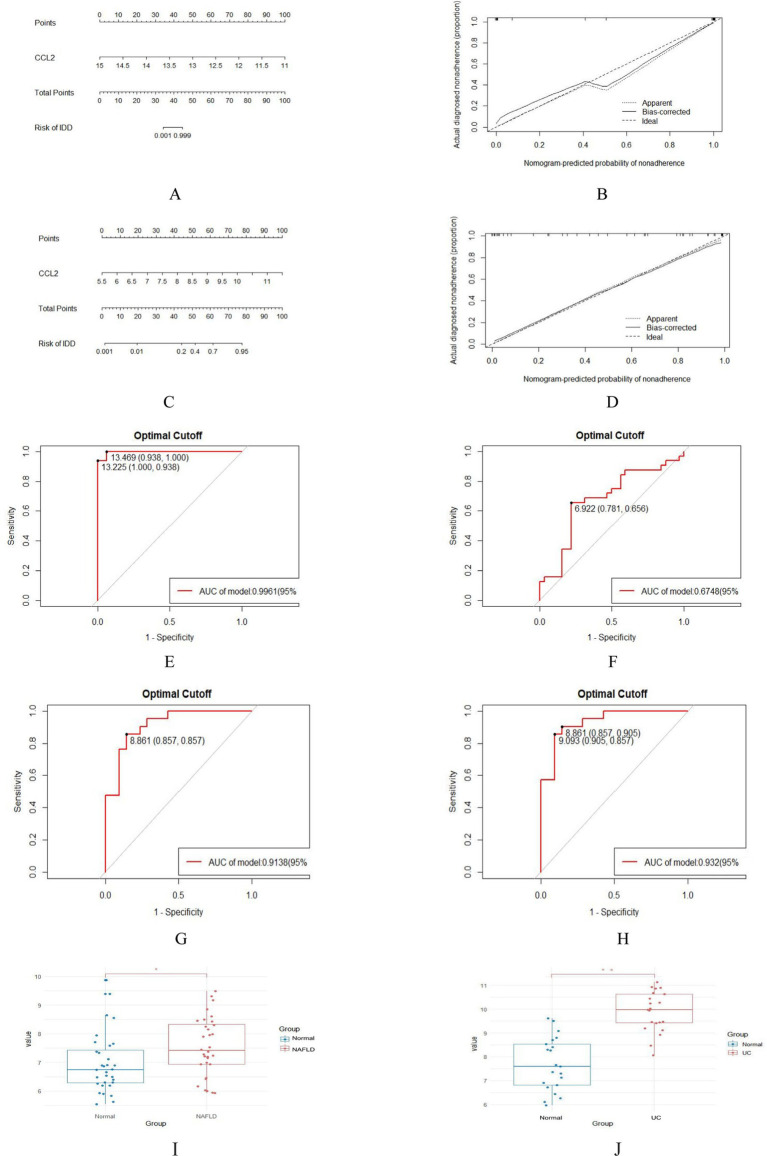
Nomogram construction and prediction accuracy evaluation. (A,C) Nomogram for diagnosing NAFLD with UC. (B,D) Calibration curves assessing the predictive accuracy of the nomograms. (E–H) ROC curves for candidate gene (*CCL2*) in the training (GSE89632 and GSE87466) and validation sets (GSE48452 and GSE92415). (I,J) Validation of key genes. *CCL2* were significantly up-regulated in NAFLD (GSE48452) and UC (GSE92415). *p < 0.05; **p < 0.01.

In order to evaluate the role of one biomarker in NAFLD and UC diagnosis, the nomogram containing one biomarker (*CCL2*) was generated (Figures 7A,C). The calibration curve generated from this nomogram revealed a slight difference between actual and predicted values. This suggests that the nomogram has a high diagnostic value (Figures 7B,D). Moreover, ROC analysis was used to determine the AUC and 95% CI of the candidate gene in the training and validation sets. The findings were as follows: *CCL2* (AUC: 0.9961, 95% CI: 0.938–1.000) in GSE89632, *CCL2* (AUC: 0.6748, 95% CI: 0.656–0.781) in GSE48452, *CCL2* (AUC: 0.9138, 95% CI: 0857–0.857) in GSE87466, *CCL2* (AUC: 0.932, 95% CI: 0.857–0.905) in GSE92415 (Figures 7E–H). In the validation set, it was observed that the expression level of *CCL2* was significantly higher than that of the normal group (Figures 7I,J). The above results show that *CCL2* has a high diagnostic value.

### 3.6 Immune cell correlation analysis

**Figure 8:**
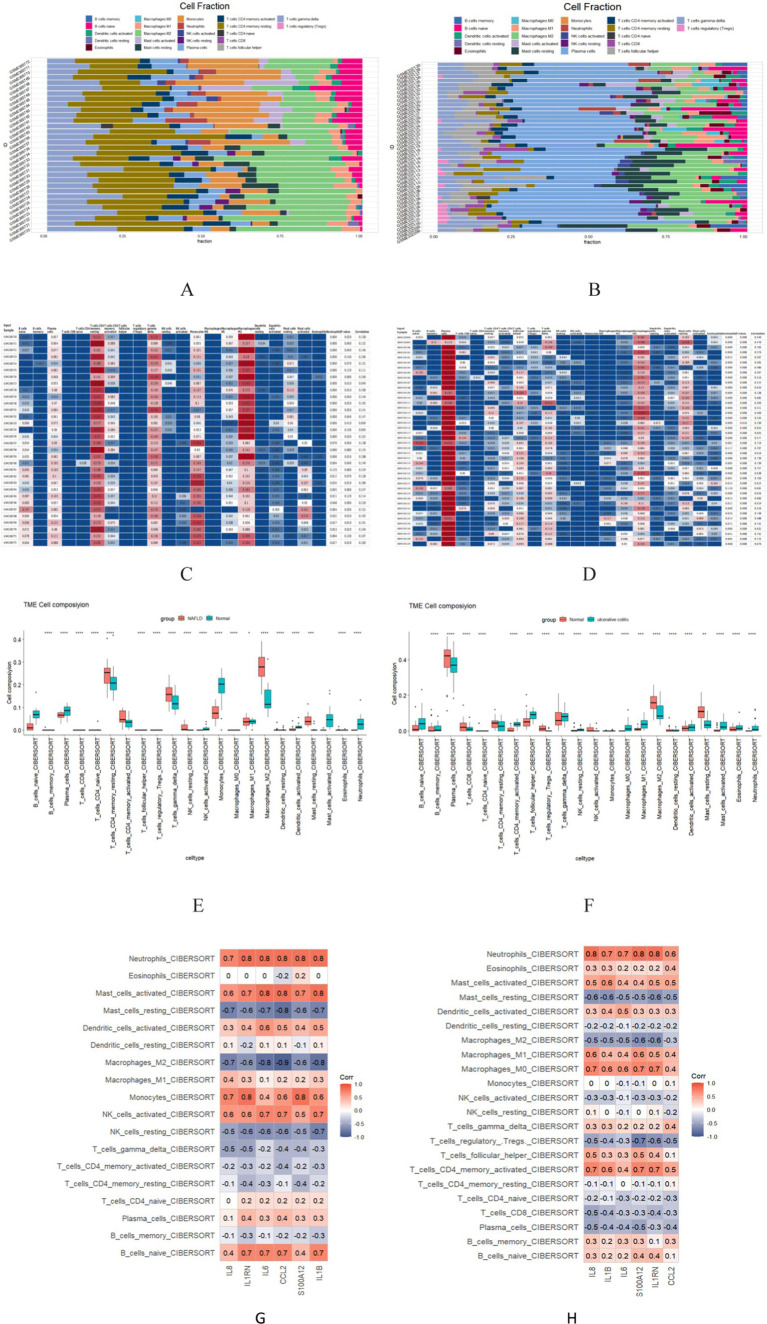
The 22 immune cells and their correlation with NAFLD and UC. (A,C,E) The distribution of 22 immune cells in NAFLD and Normal samples is depicted via the Scale diagram, the heatmap, and the Multiple sets of box diagram. (B,D,F) The distribution of 22 immune cells in samples with HC and UC is depicted by the Scale diagram, the heatmap, and the Multiple sets of box diagram. (G) Spearman correlation analysis of the 6 common core genes and 22 immune cells in NAFLD. (H) Spearman correlation analysis of the 6 common core genes and 22 immune cells in UC. *p < 0.05; **p < 0.01.

The enrichment analysis results revealed a significant overlap between the genes associated with NAFLD and UC, particularly in terms of their involvement in inflammatory response and immune regulation. Consequently, a more in-depth analysis of immune infiltration is required for both NAFLD and UC. First, Both CIBERSORTx and CIBERSORT algorithms were used to evaluate the proportions of 22 immune cells in GSE89632 (16 normal controls and 16 patients with NAFLD) and GSE87466 (21 normal controls and 21 patients with UC). The proportions of the immune cell composition showed clustering and individual differences (Figures 8A–D). T cells CD4 memory resting, Mast cells restings, Tγδ, Macrophagess M1, and Macrophagess M2 were significantly upregulated in NAFLD samples; however, the levels of Plasma cells, Monocytes, and NK cells activated were significantly decreased. In UC samples, T cells CD4 memory activated, T cells follicular helper and Macrophagess M1 were significantly upregulated; however, the levels of Plasma cells, T cells CD8, and Macrophagess M2 were significantly decreased (Figures 8E,F). Additionally, our analysis identified feature genes closely associated with immune cell infiltration. In NAFLD samples, CCL2 was positively correlated with monocytes and mast cells activated; however, it was negatively correlated with mast cells resting and macrophage M2 (Figure 8G). In UC samples, CCL2 was positively correlated with Neutrophils, and Macrophagess M0 negatively correlated with mast cells resting and Macrophagess M2 (Figure 8H). These genes likely play a critical role in shaping the local microenvironment within both NAFLD and UC-affected tissues.

## 4 Discussion

The enterohepatic axis is thought to play a vital role in the comorbidities of NAFLD and UC. Recent studies have shown that the interaction between the gut microbiota and the host influences the development and course of both diseases through multiple pathways (22). In addition, metabolic syndrome is commonly seen in NAFLD patients and is characterized by insulin resistance, obesity, etc. These factors may also influence the development of UC by altering microbiome composition (23). Therefore, based on the physiological mechanisms of the hepatoenteric axis of these two diseases, a deeper understanding of these complex interactions through bioinformatics approaches could help to shed light on the pathogenesis of the coexistence of NAFLD and UC and provide potential targets for the development of new therapeutic strategies.

The results of the GO enrichment analysis indicate that key genes are primarily enriched in inflammatory response, chemotaxis, and taxis. This suggests that inflammatory processes play a significant role in the co-pathogenesis of NAFLD and UC. Oxic lipid entities such as palmitic acid, oxLDL, and free cholesterol, which are increased in NAFLD, can activate innate immune pathways (e.g., TLR4, IL-1β, TNFα, NFκB) to drive hepatic inflammation (24). Pro-inflammatory cytokines such as TNFα, IL-6, IL-12, and IL-23 play a crucial role in the occurrence and progression of UC. These cytokines are closely associated with the inflammatory response seen in UC patients (25). One important factor contributing to this inflammatory response is the increased permeability of the intestinal barrier. When the intestinal barrier becomes more permeable, immune cells are exposed to higher levels of PAMPs. This exposure triggers an inflammatory response and allows harmful substances, such as bacterial toxins or inflammatory molecules, to enter systemic circulation more easily, which may provide a beneficial way to initiate and further develop NAFLD (25, 26). In the GSEA and KEGG analysis, we find that the IL-17 and TNF signaling pathways have emerged as crucial players in the development and progression of two diseases. One key finding is that IL-17, a pro-inflammatory cytokine, is pivotal in promoting the transition from NAFL to NASH (27). Research has shown that the IL 23/IL17 axis is critically involved in UC pathogenesis. The interaction between these two molecules promotes the differentiation and activation of Th17 cells, which contribute significantly to immune responses and inflammation (28). Additionally, the TNF signaling pathway has been implicated in both NAFLD and UC pathogenesis based on numerous pieces of evidence (29, 30).

Furthermore, we used the Cytoscape plugin cytoHubba to score and screen 34 genes. This analysis identified the top 10 key genes: IL1B, IL6, CXCL8 (IL8), CCL2, S100A12, IL1RN, S100A9, CCL3, CD44, and S100A8. These genes are believed to play crucial roles in NAFLD and UC. IL1B, a pro-inflammatory cytokine, has been found to induce the formation of lipid droplets in hepatocytes and promote the recruitment of neutrophils in the liver. Additionally, IL1B contributes to the progression from liver inflammation to liver fibrosis (31). Patients with UC have increased levels of IL1B mRNA in their intestine tissues. This increase reduces occludin expression by enterocytes and increases TJ permeability. Consequently, it promotes the occurrence and development of UC. Another cytokine, IL-6, has been implicated in liver injury and apoptosis when overexpressed for long periods (32). Inhibiting IL-6 is an effective treatment for UC (33). CCL2 signaling is associated with metabolic disorders during the development of NASH and contributes to lipid accumulation in hepatocytes (34). The CXCL8-CXCR1/2 axis participates in UC pathogenesis through multiple signaling pathways, including PI3k/Akt, MAPKs, and NF-κB (35). Based on these findings, these genes, IL1B, IL-6, CCL2, and CXCL8, have been shown to contribute not only to NAFLD but also to UC occurrence and development. They hold potential as new treatment targets.

One diagnostic marker was obtained from 24 core genes using two algorithms to investigate the diagnostic markers of NAFLD complicated by UC. Numerous studies have indicated that Chemokines are a group of signaling proteins that play crucial roles in various biological processes involved in the development and progression of NAFLD. These processes include inflammation, immune cell migration, and inflammatory mediator secretion. By regulating chemotaxis, which is the movement of immune cells toward sites of inflammation, chemokines contribute to the overall pathophysiology of NAFLD (36, 37). Conversely, genetic depletion or pharmacological inhibition of CCL2 in mice has been shown to improve steatosis progression, alleviate hepatic inflammatory response, and reduce liver injury (38, 39). Another study has shown that the expression level of CCL2 in the liver positively correlates with the severity of fatty liver disease. Elevated CCL2 levels may promote the migration and infiltration of monocytes, thereby exacerbating liver inflammation and fibrosis processes (40–42). Moreover, CCL2 is crucial in recruiting macrophages during the inflammatory process (43). Zheng et al. improve the inflammatory status of the liver by suppressing CCL2 expression (44). Therefore, these findings suggest that the CCL2 gene may serve as a biomarker for NAFLD, offering new avenues for early screening and intervention. Similarly, in the study of the etiology and pathogenesis of UC, the role of the CCL2 gene has attracted significant attention. Previous studies have shown that CCL2 expression levels are associated with various inflammatory diseases, including UC (45). CCL2 is a potent chemotactic cytokine that controls the chemotaxis and infiltration of monocytes/macrophages in the gut (46). CCL2 enhances the expression and production of other inflammatory cytokines and the infiltration of inflammatory cells and macrophages (47). Previous data suggest that ulcerative colitis is improved by inhibiting the CCL2/NF-κB/IL-18 pathway, thereby preserving colon function (45). In addition, studies by Yamada et al. (48) have found that palmitic acid, a long-chain fatty acid produced by intestinal flora, can promote the increase of CCL2 secretion, leading to a significant increase in the number of macrophages in the liver and aggravating high-fat diet-induced mouse steatosis. However, this change will be reversed after the removal of intestinal flora. These results suggest that some metabolites of intestinal flora can affect the process of NAFLD through macrophages. Therefore, CCL2 may be an important gene in the comorbidities of NAFLD and UC, mainly promoting the occurrence of the comorbidities of NAFLD and UC by inducing inflammation on the physiological basis of enterohepatic axis.

There are immune cells in the liver that play a crucial role in developing NAFLD (49). On the other hand, UC is an autoimmune disease caused by an overactive immune system (50). To better understand how immune cell infiltration affects NAFLD with UC, we assessed immune infiltration using CIBERSORT. Previous studies have shown that dysregulation of M1/M2 macrophages can lead to chronic inflammation, cancer, and NAFLD (51). Increasing evidence suggests that γΔ T cells in the liver respond to liver-targeted injury and regulate the progression of liver disease (52). The dysfunction of CD4+ T cells has emerged as a significant pathological factor in the advancement of NAFLD and NASH. Both human and mouse NASH models have revealed peripheral and intrahepatic CD4+ T cell accumulation (53). Additionally, macrophages are essential effector cells of the innate immune system that contribute to intestinal mucosal homeostasis (54). Previous research has indicated potential therapeutic benefits from targeting macrophages in UC treatment (55). CD4+ T cells consist of various functionally diverse subsets such as Th1, Th2, Th17, and Treg cells. All these subsets have been implicated in initiating or propagating intestinal inflammation in UC patients (56).

The correlation between the six hub genes and immune cells revealed consistent expression of these genes with multiple immune cells. For instance, all six hub genes were overexpressed in NAFLD patients compared to HCs. Additionally, the expression of these genes was positively correlated with an increase in resting mast cells, which were found to be elevated in NAFLD patients compared to HCs. Based on this information, the high expression of these hub genes may contribute to the increase in multiple immune cells associated with NAFLD. Furthermore, these genes may play a role in shaping the characteristic immune microenvironment of NAFLD. Our research suggests that the CCL2 gene may be an essential core gene among these six identified genes. CCL2 is a chemokine that attracts inflammatory monocytes to stressed or injured tissues. Infiltrating inflammatory monocytes and upregulation of CCL2 have been strongly implicated in liver disease pathogenesis based on animal models (57, 58). Similarly, studies have shown that macrophages can secrete chemokines and mediate immune cell recruitment during the inflammatory cascade process (59). The FBXW7/EZH2/CCL2/CCL7 pathway has been shown to exacerbate colitis severity by recruiting CX3CR1 proinflammatory MPhs (60).

In summary, in this study, we explored the potential mechanisms of comorbidities between NAFLD and UC based on bioinformatics analysis. The results show that inflammation plays a vital role in the co-pathogenesis of NAFLD and UC, primarily through inflammatory response, immune regulation, and other pathways, affecting both pathological processes. We found that a critical diagnostic gene, CCL2, is abnormally expressed in both NAFLD and UC, suggesting that this molecule may be a common pathogenic factor for both diseases. Through in-depth analysis of related genes and signaling pathways, we propose that the dual role of inflammatory factors such as CCL2 in NAFLD and UC may provide new targets for future diagnosis and treatment. However, there are some limitations. First, the study data came from an existing database, so the authenticity and completeness of the results depend on the data. Second, the results do not reflect all actual cellular network characteristics in living organisms, and our findings still need to be validated *in vivo* and *in vitro* to guide clinical practice better.

## 5 Conclusion

In this study, we have identified 34 key genes that are associated with the comorbidity between NAFLD and UC, which were mainly involved in the IL-17 signaling pathway. 10 hub genes (including *IL1B*, *IL6*, *CXCL8 (IL8)*, *CCL2*, *S100A12*, *IL1RN*, *S100A9*, *CCL3*, *CD44*, and *S100A8*) may have a significant impact on the pathophysiological mechanisms of these two diseases. The machine learning analysis also revealed a single feature gene *CCL2* that could potentially serve as a diagnostic biomarker for NAFLD and UC. This diagnostic marker not only affects immune cells, but also has the potential to become a therapeutic target when NAFLD coexists with UC.

## Data Availability

Publicly available datasets were analyzed in this study. This data can be found at: GSE89632, GSE87466, GSE48452, and GSE92415.

## Footnotes

^1^ http://cibersort.stanford.edu/
